# Supplementary material for: Tumor Promoting Effect of BMP Signaling in Endometrial Cancer
Source: Int J Mol Sci. 2021 Jul 23;22(15):7882. doi: 10.3390/ijms22157882 (PMC8346149; doi:10.3390/ijms22157882)
Supplement: Supplementary file 1 [file ijms-22-07882-s001.zip › ijms-1279578-supplementary.pdf]

## Supplementary Materials

## Table S1. Antibodies used in the present study.

| antibody | species | clonality | dilution | source | catalogue number |
|---|---|---|---|---|---|
| phospho-SMAD1/5/8 | Rabbit | polyclonal | 1:1000 | Cell Signaling Technology | 9511 |
| vimentin | Rabbit | monoclonal | 1:100(IF), 1:1000(WB) | Cell Signaling Technology | 5741 |
| c-KIT | Rabbit | monoclonal | 1:1000 | Cell Signaling Technology | 3074 |
| E-cadherin | Rabbit | monoclonal | 1:1000(WB) | Cell Signaling Technology | 3195 |
| TWSG1 | Rabbit | polyclonal | 1:1000 | Abcam | ab183890 |
| α -tubulin | Mouse | monoclonal | 1:1000 | Abcam | ab7291 |
| SMAD1 | Rabbit | polyclonal | 1:1000 | Abcam | ab63356 |
| E-cadherin | Rabbit | monoclonal | 1:100(IF) | Abcam | ab1416 |
| N-cadherin | Mouse | monoclonal | 1:1000 | BD biosciences | 610920 |
| secondary Rabbit | Goat |  | 1:5000 | Invitrogen | 31460 |
| secondary Mouse | Goat |  | 1:5000 | Invitrogen | 31430 |
| Alexa Fluor 488 Rabbit | Goat |  | 1:200 | Invitrogen | A32731 |

**Table S2. Primer pairs used in the present study.**

| Gene | 5' primer sequence | 3' primer sequence |
|---|---|---|
| GAPDH | GGAGTCAACGGATTTGGTCGTA | GGAGTCAACGGATTTGGTCGTA |
| ACVRL1 | ATCTGAGCAGGGCGACAGC | ACTCCCTGTGGTGCAGTCA |
| ACVR1 | CATGGCCCCCGAAAGTTCTTGATGA | GCCACCTCCCACAAGACAAGTCCAAA |
| BMPR1A | TTTATGGCACCCAAGGAAAG | TGGTATTCAAGGGCACATCA |
| BMPR1B | AGCAGTGATGAGTGTCTAAGGC | AGCCTTGATGCAGGATTGTGA |
| ACVR2A | GCCACAAACCCGCCATATCTCACA | TGCCAGCCTCAAACTTTAACGCCAA |
| ACVR2B | CAACTGCTACGATAGGCAGGA | CGTTGCAGAAGTTGCCTTCA |
| BMPR2 | GGCTGAACTTATGATGATTTGGGAA | CACGCCTATTATGTGACAGGTTGC |
| ID1 | AGCACGTCATCGACTACATCAGG | GGATTCCGAGTTCAGCTCCAA |
| CD44 | GGGACTTTGCCTCTTGCAGTT | CGGCAGGTTACATTCAAATCG |
| c-KIT | CACCGAAGGAGGCACTTACACA | TGCCATTCACGAGCCTGTCGTA |
| SNAIL | TGCCCTCAAGATGCACATCCGA | GGGACAGGAGAAGGGCTTCTC |
| SLUG | ATCTGCGGCAAGGCGTTTTCCA | GAGCCCTCAGATTTGACCTGTC |
| NBL1 | TCCACAGAGTCCCTGGTTCACT | GCTACAGTGCAGGATCTTCTCC |
| DAND5 | TAAGGCTGTGCCCTTCGTTCAG | AACGCTTGCGAGCAGGCATACA |
| CER1 | CAGGACAGTGCCCTTCAGCCA | ACAGTGAGAGCAGGAGGTATGG |
| GREM1 | TCATCAACCGCTTCTGTTACGGC | CAGAAGGAGCAGGACTGAAAGG |
| GREM2 | AGAGTGACTGGTGCAAGACGCA | GGATGTAGAAGGAGTTGCACTGG |
| NOG | GCCAGCACTATCTCCACATCCG | AGCAGCGTCTCGTTCAGATCCT |
| CHRD | TCACGCTGCTAGGAAATGGCTC | TGAGGCTTGGTCTCCAGTGTCA |
| TWSG1 | CTTTGGGACGAGTGCTGTGACT | GAGAAGGGATCGGTTCATGCAG |
| SOSTDC1 | TCAAGCCAGAAATGGAGGCAGG | GCCATCAGAGATGTATTTGGTGG |
| BMPER | GTGAAGAGTGCCTCCTACGAGT | GTCCTGCCTTTCACACAAGCAC |

Fig. S1

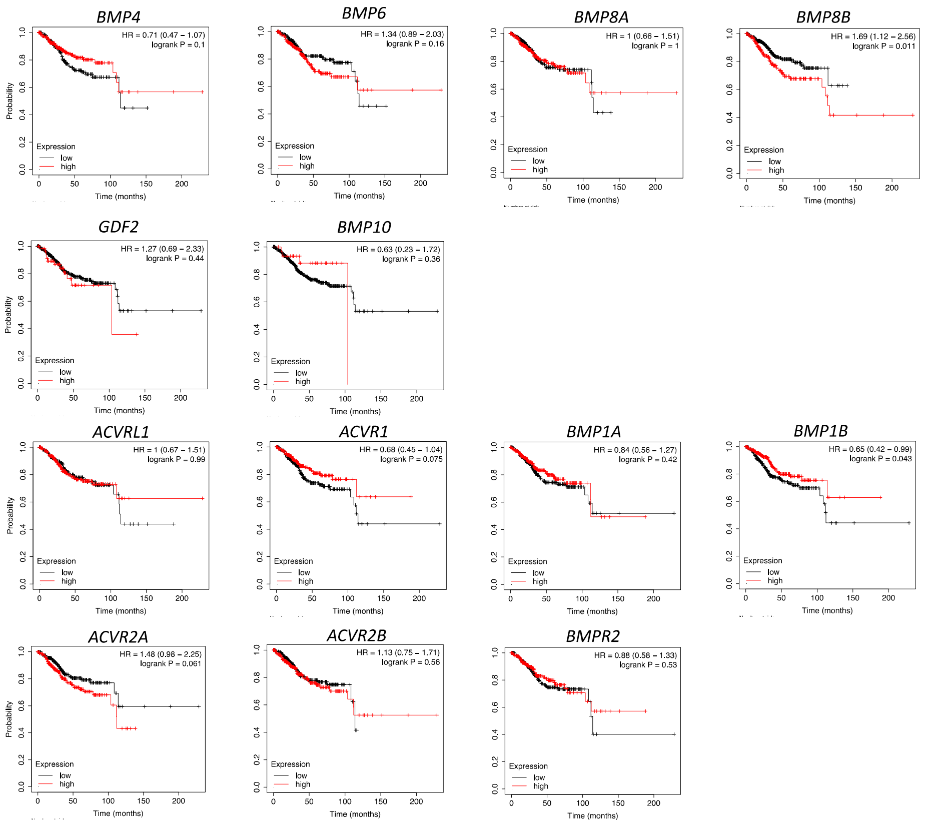


**Fig. S1. Correlation between mRNA expressions of BMP ligands and receptors and overall survival in 542 EC patients.**

Overall survival was analyzed using RNA-Seq data of KM plotter, which contained 542 EC patients. Patients were divided into two groups, i.e. above or below median mRNA expression.

Fig. S2

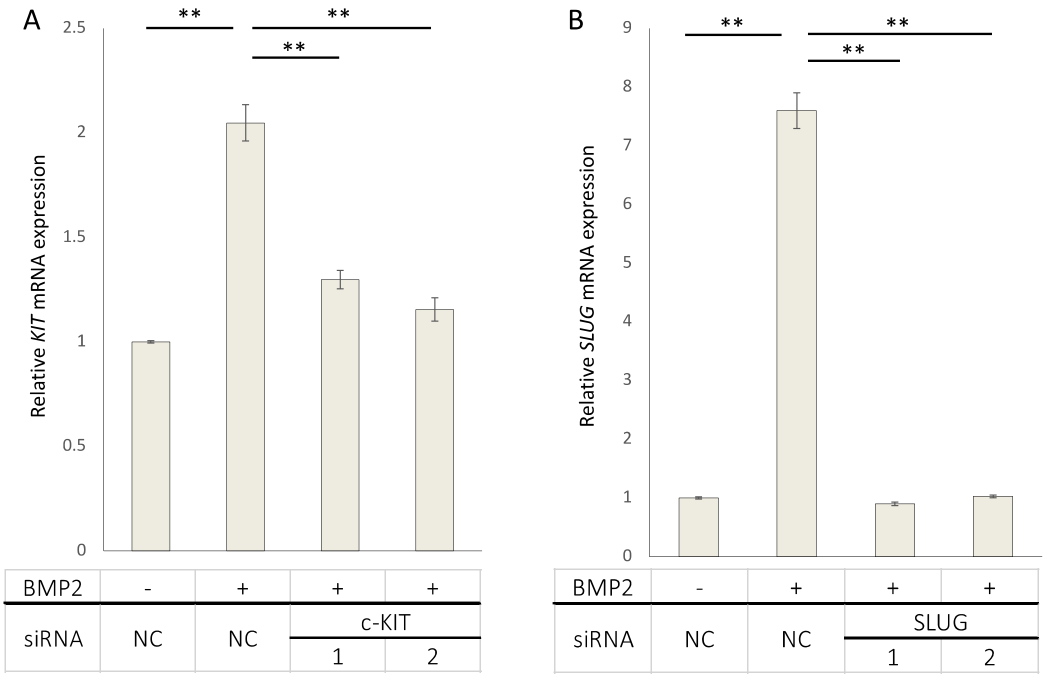


**Fig. S2. Efficiency of c-KIT and SLUG siRNA knockdown in Ishikawa cells.**

(A) Ishikawa cells were transfected with siNC, siKIT-1 or siKIT-2; after 48 h, cells were incubated in the presence or absence of 20 ng/ml BMP2 in 1% FBS-containing medium for an additional 72 h. *KIT* mRNA expression was evaluated with qRT-PCR. (B) Ishikawa cells were transfected with siNC, siSLUG-1, or siSLUG-2 for 48 h; cells were then incubated in the presence or absence of 20 ng/ml BMP2 in 1% FBS-containing medium for an additional 2 h. *SLUG* mRNA expression was evaluated with qRT-PCR. mRNA expression was normalized relative to CT. The results in panel A and B are shown as the mean ± SE. ** *P*-value < 0.01.

Fig. S3

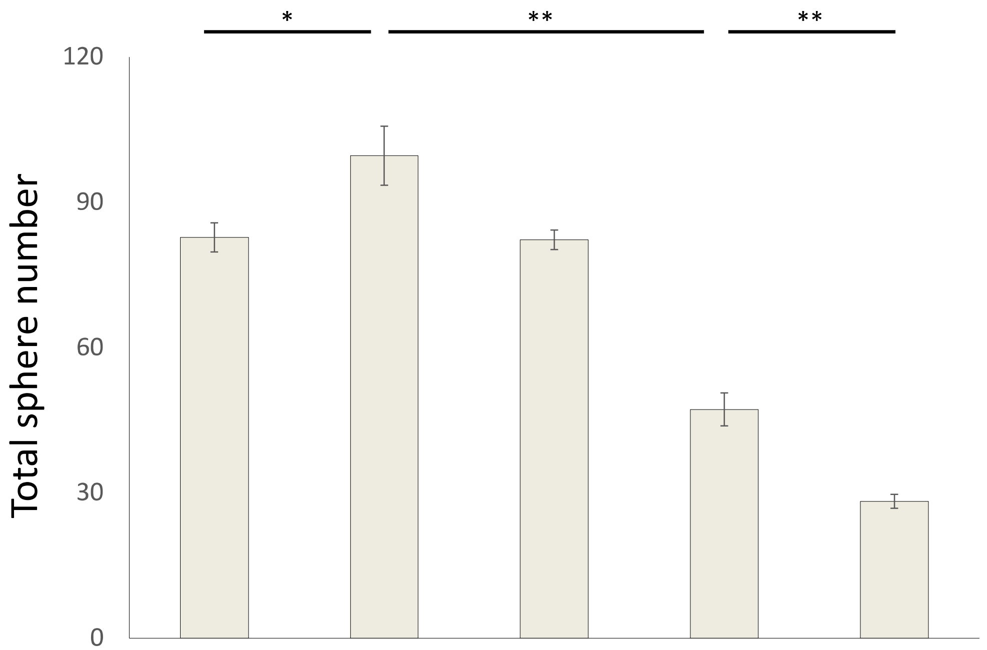


| | | | | | |
|---|---|---|---|---|---|
| BMP2 | - | + | + | + | + |
| LDN | - | - | + | - | + |
| CBDCA | - | - | - | + | + |

**Fig. S3. LDN193189 augments the inhibitory effect of carboplatin on the BMP2-induced sphere formation.**

Sphere formation assay was performed using Ishikawa cells in the absence (CT) or presence of 200 nM LDN193189, 25 μM carboplatin (CBDCA), and 20 ng/ml BMP2 for 8 days, whereafter the number of spheres was counted. The results are shown as the mean ± SE. * *P*-value < 0.05, ** *P*-value < 0.01.
